# Basal position of two new complete mitochondrial genomes of parasitic Cymothoida (Crustacea: Isopoda) challenges the monophyly of the suborder and phylogeny of the entire order

**DOI:** 10.1186/s13071-018-3162-4

**Published:** 2018-12-10

**Authors:** Cong J. Hua, Wen X. Li, Dong Zhang, Hong Zou, Ming Li, Ivan Jakovlić, Shan G. Wu, Gui T. Wang

**Affiliations:** 1 Key Laboratory of Aquaculture Disease Control, Ministry of Agriculture, and State Key Laboratory of Freshwater Ecology and Biotechnology, Institute of Hydrobiology, Chinese Academy of Sciences, Wuhan, 430072 People’s Republic of China; 2 University of Chinese Academy of Sciences, Beijing, 100049 People’s Republic of China; 3 Bio-Transduction Lab, Biolake, Wuhan, 430075 People’s Republic of China

**Keywords:** *Ichthyoxenos japonensis*, *Tachaea chinensis*, Mitochondrial genome, RNA secondary structure, Gene rearrangement, Phylogenetic analysis

## Abstract

**Background:**

Isopoda is a highly diverse order of crustaceans with more than 10,300 species, many of which are parasitic. Taxonomy and phylogeny within the order, especially those of the suborder Cymothoida Wägele, 1989, are still debated. Mitochondrial (mt) genomes are a useful tool for phylogenetic studies, but their availability for isopods is very limited. To explore these phylogenetic controversies on the mt genomic level and study the mt genome evolution in Isopoda, we sequenced mt genomes of two parasitic isopods, *Tachaea chinensis* Thielemann, 1910 and *Ichthyoxenos japonensis* Richardson, 1913, belonging to the suborder Cymothoida, and conducted comparative and phylogenetic mt genomic analyses across Isopoda.

**Results:**

The complete mt genomes of *T. chinensis* and *I. japonensis* were 14,616 bp and 15,440 bp in size, respectively, with the A+T content higher than in other isopods (72.7 and 72.8%, respectively). Both genomes code for 13 protein-coding genes, 21 transfer RNA genes (tRNAs), 2 ribosomal RNA genes (rRNAs), and possess a control region (CR). Both are missing a gene from the complete tRNA set: *T. chinensis* lacks *trnS*1 and *I. japonensis* lacks *trnI*. Both possess unique gene orders among isopods. Within the CR of *I. japonensis* (284 bp), we identified a repetitive region with four tandem repeats. Phylogenetic analysis based on concatenated nucleotide sequences of 13 protein-coding genes showed that the two parasitic cymothoids clustered together and formed a basal clade within Isopoda. However, another parasitic cymothoid, *Gyge ovalis* Shiino, 1939, formed a sister group with the suborder Limnoriidea Brandt & Poore in Poore, 2002, whereas two free-living cymothoid species were located in the derived part of the phylogram: *Bathynomus* sp. formed a sister group with the suborder Sphaeromatidea Wägele, 1989, and *Eurydice pulchra* Leach, 1815 with a clade including *Bathynomus* sp., Sphaeromatidea and Valvifera G. O. Sars, 1883.

**Conclusions:**

Our results did not recover the suborders Cymothoida and Oniscidea Latreille, 1802 as monophyletic, with parasitic and free-living cymothoidans forming separate clades. Furthermore, two parasitic cymothoidans formed the sister-clade to all other isopods, separated from Epicaridea Latreille, 1825, which challenges currently prevalent isopod phylogeny. Additional mt genomes of parasitic and free-living isopods might confer a sufficient phylogenetic resolution to enable us to resolve their relationships, and ultimately allow us to better understand the evolutionary history of the entire isopod order.

**Electronic supplementary material:**

The online version of this article (10.1186/s13071-018-3162-4) contains supplementary material, which is available to authorized users.

## Background

Within Crustacea, the isopods form a diverse group (10,300 species) [1] comprising 11 recognized suborders [2]. They are common around the globe, with habitats ranging from deep seas to mountains, even including arid deserts [3, 4]. Species of the suborder Cymothoida Wägele, 1989 inhabits both freshwater and marine habitats (most diverse in the tropics), and includes mobile predators, scavengers and all isopods parasitizing on fishes and other crustaceans [5, 6]. Among other orders relevant for this study, members of the Valvifera G. O. Sars, 1883 and Sphaeromatidea Wägele, 1989 are benthic, and most species are detritivores [5]. Species of the Asellota Latreille, 1802, which occur both in freshwater and marine habitats, are the predominant deep-sea isopod taxon [7]. Species of the Limnoriidea Brandt & Poore in Poore, 2002 are herbivores, with the largest family, the Limnoriidae White, 1850, comprising predominately tropical borers feeding on wood [5, 7]. The Oniscidea Latreille, 1802 contains most of the terrestrial isopod species [8]. The classification of the Isopoda has been continuously studied and revised over the years; for example, the former suborder Flabellifera Sars, 1882 has been divided into two suborders, the Cymothoida and the Sphaeromatidea, but the Isopoda remains poorly resolved [4, 9]. A majority of morphology-based studies have agreed that the Phreatoicidea Stebbing, 1893, Asellota and Oniscidea are the basal isopod groups, whereas the Cymothoida is generally regarded as the most derived group [8, 10–16]. However, some analyses, based on nuclear (*18S*) and mitochondrial datasets [17, 18], resolved the Phreatoicidea as a derived clade, rather than a basal one, whereas parasitic Cymothoida comprised the basal branch. Monophyly of parasitic Cymothoida was also questioned by some morphological evidence [19]. In addition, the phylogenetic position of the infraorder Epicaridea Latreille, 1825 within the Cymothoida (and Isopoda) is controversial [5, 10, 16, 20]. Therefore, although the phylogenetic relationships within the Cymothoida have been revised [21] and discussed extensively, they remain far from resolved.

Parasitic isopods were traditionally assigned to three superfamilies, the Bopyroidea Rafinesque, 1815, the Cryptoniscoidea Kossmann, 1880 and the Cymothooidea Leach, 1814 [6]. The Cymothooidea includes ten families [2], which exhibit a progressive gradient from commensal predation and micropredation towards parasitism, culminating with the Cymothoidae Leach, 1818, with all species being obligatory parasites of fishes [3–5]. Due to problems in data collection and species identification, as well as the paucity of studies and specialists for this taxon, the large family Cymothoidae is still widely regarded as taxonomically the least understood and the most ‘troublesome’ isopod group [4, 22]. Studies of phylogenetic relationships within the Cymothoidae have been conducted using morphological [22, 23], zoogeographical [4, 22, 24], and ecological data, such as the host species [4, 6, 25] and site of attachment on the host [4, 22, 26]. However, relationships within the Cymothoidae are still not resolved, especially in relation to the question of the evolution of parasitic life-styles [4].

Incomplete or brief morphological descriptions available for some isopod species may result in synonymies; for example, a study of ten recognized species of *Ichthyoxenos* Herklots, 1870 in China concluded that they should all be synonymized with *I. japonensis* Richardson, 1913 [27]. Therefore, the taxonomy of this genus and its relationships to other similar genera (e.g. *Elthusa* Schioedte & Meinert, 1884 and *Mothocya* Costa, in Hope, 1851) remain poorly resolved [28, 29]. In conclusion, more molecular data and a comprehensive revision are urgently needed to resolve classification and identification of species belonging to *Ichthyoxenos*.

The use of complete mitochondrial (mt) genomes is becoming increasingly important for phylogenetic reconstruction [30–33]. Nucleotide and amino acid sequences, strand-specific nucleotide bias [30], tRNA secondary structures [34], as well as gene rearrangements [30] have been used for phylogenetic inference. In contrast to the 269 complete crustacean mt genomes currently (March 2018) available on GenBank, the number of complete isopod mt genomes appears strikingly small: *Bathynomus* sp. [35], *Eophreatoicus* sp. [36], *Gyge ovalis* Shiino, 1939 [37], *Ligia oceanica* Linnaeus, 1767 [38] and *Limnoria quadripunctata* Holthuis, 1949 [39]. Along with these five, ten incomplete (verified) mt genomes are also currently available (March 2018).

As parasitic isopods were not represented on GenBank when we began this study (the first mitogenome of a parasitic isopod, *G. ovalis* [37], was published as we were finishing writing the paper), the aim of our study was to sequence the complete mitogenomes of two parasitic isopods belonging to two different cymothoid families: *Tachaea chinensis* Thielmann, 1910 (Corallanidae), an ectoparasite that attaches to the ventral thoracic region of many freshwater shrimp species [40, 41], and *Ichthyoxenos japonensis* Richardson, 1913 (Cymothoidae) that parasitises the body cavity of freshwater fish [27, 42–45]. As the family Corallanidae Hansen, 1890 is generally regarded as a sister group to all remaining parasitic taxa within the suborder Cymothoida [5, 11, 25, 46, 47], the availability of these two mt genomes might help resolve questions of the identity of the basal isopod group and the monophyly of the suborder Cymothoida.

## Methods

### Sample collection and identification

*Tachaea chinensis* was collected from the body surface of a freshwater shrimp (*Macrobrachium* sp.) obtained from a fish market in Wuhan, China. *Ichthyoxenos japonensis* was collected from the body cavity of a goldfish (*Carassius auratus*) obtained in the Baihe River, Nanyang, China. *Ichthyoxenos japonensis* was morphologically identified according to the available literature [43, 48]; *T. chinensis* was identified according to the morphological traits [49], and its identity verified using the complete *16S* rDNA sequence (Additional file 1: Figure S1). Total genomic DNA was extracted from a single specimen using SDS/Proteinase K using TIANamp Genomic DNA kit (Tiangen Biotech, Beijing, China) following the manufacturer’s protocol. Voucher specimens for both species (*T. chinensis* and *I. japonensis*) are permanently stored in absolute ethanol under accession numbers IHB20160505001 and IHB20150714001, respectively, in the Key Laboratory of Freshwater Ecology and Biotechnology, Institute of Hydrobiology, Chinese Academy of Sciences, Wuhan, China. Photographs of *T. chinensis* and *I. japonensis* are provided in Additional file 2: Figure S2 and Additional file 3: Figure S3, respectively.

### Mt genome amplification and sequencing

We designed primers (Additional file 4: Table S1) according to conserved regions of mitochondrial genes in other available isopod mitogenomes, and then used these (amplified and sequenced) fragments to design specific primers for amplification of the complete mt genomes (Additional file 4: Table S1). PCR reactions were conducted in a 20 μl reaction mixture, containing 7.4 μl double-distilled water, 10 μl 2× PCR buffer (Mg^2+^, dNTP plus; Takara, Dalian, China), 0.6 μl of each primer, 0.4 μl rTaq polymerase (250 U, Takara) and 1 μl DNA template. Amplification was performed under the following conditions: initial denaturation at 98 °C for 2 min; followed by 40 cycles at 98 °C for 10 s, 48–60 °C for 15 s, 68 °C for 1 min/kb; and a final extension at 68 °C for 10 min. PCR products were sequenced (Sanger) bidirectionally at Sangon Company (Shanghai, China).

### Mt genome assembly, annotation and analysis

The complete mt sequences were assembled manually and aligned against other published mt genome sequences of isopods using the program MAFFT 7.149 [50] to determine the gene boundaries. BLAST and ORF Finder NCBI tools were also used to identify and annotate the protein-coding genes (PCGs) and rRNAs. The boundaries of the two rRNAs were tentatively identified *via* sequence comparison with the published isopod mt rRNAs. Transfer RNA genes and their secondary structures were identified using tRNAscan-SE 1.21 [51] and ARWEN 1.2 [52]. Secondary structures of the rRNAs and the CR were predicted using Mfold software [53]. Tandem repetitive elements were detected with Tandem Repeats Finder software [54]. Nucleotide composition (%) of each gene, non-coding region and the complete mt genome were calculated using DNASTAR’s Lasergene sequence analysis software [55]. Nucleotide compositions of different regions, codon usage and relative synonymous codon usage (RSCU) values of PCGs were analyzed with MEGA v.6.0 software [56].

### Phylogenetic analysis

Phylogenetic analysis was carried out using the two mitogenomes sequenced for this study, as well as 15 isopod mitogenomes (5 complete and 10 partial) available on GenBank (March 2018). To facilitate the comparison with previous phylogenetic analyses based on the mt genomes of isopods, following the methodology adopted by Shen et al. [35] and Yu et al. [37], we also used six decapod species as the outgroup: *Alvinocaris longirostris* Kikuchi & Ohta, 1995 (GenBank: AB821296) [57], *Austinograea rodriguezensis* Tsuchida & Hashimoto, 2002 (GenBank: JQ035658) [58], *Geothelphusa dehaani* White, 1847 (GenBank: AB187570) [59], *Halocaridina rubra* Holthuis, 1963 (GenBank: KF437508) [60], *Panulirus japonicus* Von Siebold, 1824 (GenBank: NC_004251) [61] and *Shinkaia crosnieri* Baba & Williams, 1998 (GenBank: NC_011013) [62]. Nucleotide sequence alignments were individually built for all 13 PCGs using MAFFT 7.149 [50], and then concatenated (10,494 nt in total) using an in-house program, MitoTool [63]. Poorly aligned positions were removed by Gblocks v.0.91b [64]. We used jModelTest 2 [65] to determine the optimal nucleotide model for phylogenetic analysis (evaluated according to the Akaike information criterion) [66]. GTR+I+G was the best-fit model, used for both maximum likelihood (ML) and Bayesian inference (BI). Bayesian inference analysis, conducted using MrBayes 3.2 [67], was used for phylogenetic reconstruction: 2,000,000 generations, four MC chains, and the trees were sampled every 1000 generations. The confidence values for the BI tree were expressed as the Bayesian posterior probabilities. The ML tree was constructed using RAxML [68], and the robustness of the phylogenetic results was tested through bootstrap analysis with 1000 replicates. Being aware of the debate regarding the usage of terms ‘basal’ and ‘derived’ [69], we use them specifically to refer to common ancestors, and not extant species when interpreting phylogenetic relationships.

## Results

### Mt genome organisation and base composition

**Fig. 1:**
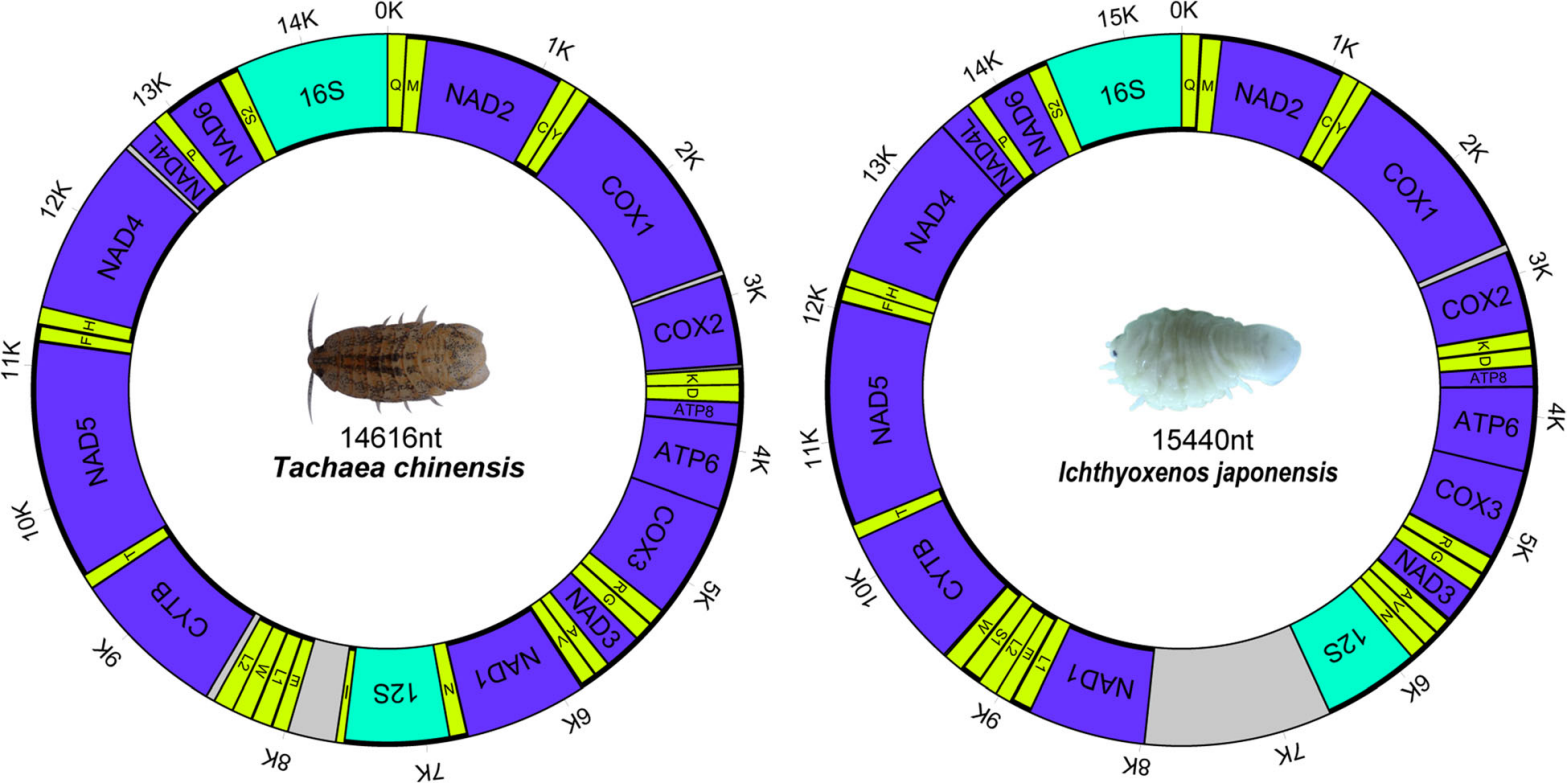
Maps of the mitochondrial genomes of *Tachaea chinensis* and *Ichthyoxenos japonensis*. The 13 protein-coding genes (blue), 21 tRNA genes (yellow) and two rRNA genes (green), as well as the control regions (grey), are depicted

The mt genomes of *T. chinensis* (GenBank: MF419232) and *I. japonensis* (GenBank: MF419233) are circular, double-stranded DNA molecules. The sizes of these two mt genomes are 14,616 bp and 15,440 bp, respectively. Both contain 36 genes, including 13 PCGs, 2 rRNAs, and 21 tRNA genes (Fig. 1). *Tachaea chinensis* lacks the *trnS1* gene and *I. japonensis* lacks the *trnI* gene. In *I. japonensis*, the heavy strand (H) encodes 23 genes and the light strand (L) encodes 13 genes (Table 1), whereas in *T. chinensis* the H and L strands encode 21 and 15 genes, respectively (Table 2). The A+T content of the two mitogenomes are 72.7% (*T. chinensis*) and 72.8% (*I. japonensis*).

**Table 1:** Gene content of the complete mitochondrial genome of *Ichthyoxenos japonensis*

Gene	Position		Size	Intergenic nucleotides	Start codon	Stop codon	Anti-codon	Strand
	From	To						
*trnQ*	1	55	55				TTG	-
*trnM*	47	108	62	-9			CAT	+
*nad*2	109	1095	987		ATT	TAA		+
*trnC*	1096	1147	52				GCA	-
*trnY*	1147	1209	63	-1			GTA	-
*cox*1	1208	2743	1536	-2	ATG	TAA		+
*cox*2	2800	3480	681	56	ATG	TAA		+
*trnK*	3476	3539	64	-5			TTT	+
*trnD*	3547	3603	57	7			GTC	+
*atp*8	3608	3754	147	4	ATA	TAA		+
*atp*6	3751	4458	708	-4	ATA	TAA		+
*cox*3	4460	5215	756	1	ATA	TAA		+
*trnR*	5208	5268	61	-8			TCG	+
*trnG*	5278	5342	65	9			TCC	+
*nad*3	5345	5694	350	2	ATC	TA		+
*trnA*	5680	5736	57	-15			TGC	+
*trnV*	5733	5792	60	-4			TAC	+
*trnN*	5790	5852	63	-3			GTT	+
*12S*	5853	6652	800					+
*NC*	6653	7971	1319					
*nad*1	7972	8907	936		ATT	TAA		-
*trnL*1	8909	8969	61	1			TAG	+
*trnE*	8960	9010	51	-10				-
*trnL*2	9011	9070	60				TAA	-
*trnS*1	9067	9132	66	-4			AGA	+
*trnW*	9126	9185	60	-7			TCA	-
*cytb*	9201	10,311	1111	15	ATG	T		-
*trnT*	10,312	10,365	54				TGT	-
*nad*5	10,365	12,062	1698	-1	ATC	TAA		+
*trnF*	12,055	12,113	59	-8			GAA	+
*trnH*	12,114	12,174	61				GTG	+
*nad*4	12,175	13,477	1303		ATA	T		-
*nad*4L	13,474	13,773	300	-4	ATT	TAA		-
*trnP*	13,774	13,831	58				TGG	-
*nad*6	13,834	14,304	471	2	ATA	TAA		+
*trnS*2	14,305	14,366	62				TGA	+
*16S*	14,367	15,440	1074					-

**Table 2:** Gene content of the complete mitochondrial genome of *Tachaea chinensis*

Gene	Position		Size	Intergenic nucleotides	Start codon	Stop codon	Anti-codon	Strand
	From	To						
*trnQ*	1	69	69				TTG	-
*trnM*	72	132	61	2			CAT	+
*nad*2	133	1149	1017		ATT	TAA		+
*trnC*	1147	1200	54	-3			GCA	-
*trnY*	1203	1264	62	2			GTA	-
*cox*1	1271	2803	1533	6	ATG	TAG		+
*cox*2	2848	3523	676	44	ATA	T		+
*trnK*	3528	3591	64	4			TTT	+
*trnD*	3592	3650	59				GTC	+
*atp*8	3648	3806	159	-3	ATA	TAA		+
*atp*6	3803	4468	666	-4	ATA	TAA		+
*cox*3	4468	5259	792	-1	ATG	TAA		+
*trnR*	5259	5319	61	-1			TCG	+
*trnG*	5315	5372	58	-5			TCC	+
*nad*3	5370	5720	351	-3	ATA	TAA		+
*trnA*	5719	5776	58	-2			TGC	+
*trnV*	5773	5837	65	-4			TAC	+
*nad*1	5852	6760	909	14	ATA	TAA		-
*trnN*	6758	6821	64	-3			GTT	+
*12S*	6822	7611	790					+
*trnI*	7612	7669	58				AAT	-
*NC*	7670	7991	322					
*trnE*	7992	8055	64				TTC	-
*trnL*1	8050	8113	64	-6			TAG	-
*trnW*	8108	8171	64	-6			TCA	-
*trnL*2	8171	8229	59	-1			TAA	-
*cytb*	8290	9417	1128	60	ATA	TAA		-
*trnT*	9420	9482	63	2			TGT	-
*nad*5	9484	11,178	1695	1	ATA	TAA		+
*trnF*	11,171	11,230	60	-8			GAA	+
*trnH*	11,221	11,282	62	-10			GTG	-
*nad*4	11,283	12,555	1273		ATG	T		-
*nad*4L	12,598	12,894	297	42	ATA	TAA		-
*trnP*	12,895	12,957	63				TGG	-
*nad*6	12,959	13,453	495	1	ATA	TAG		+
*trnS*2	13,444	13,503	60	-10			TGA	+
*16S*	13,504	14,616	1113					-

### Protein-coding genes and codon usage

The total length of the concatenated 13 PCGs of *T. chinensis* and *I. japonensis* was 10,956 bp and 10,950 bp, respectively (minus the stop codons). All PCGs of *T. chinensis* and *I. japonensis* start with the typical ATN start codons: ATA, ATC, ATG and ATT. The PCGs use TAA and TAG as termination codons, with the exception of the *nad*4 gene of both species, *cob* and *nad*3 genes of *I. japonensis*, and *cox*2 of *T. chinensis*, which use an incomplete termination codon (T-- or TA-). The incomplete termination codons, commonly found in metazoan mt genomes, are believed to be completed by mRNA polyadenylation [70]. Codon usage, relative synonymous codon usage (RSCU) and codon family proportion (corresponding to the amino acid usage) of the two parasitic isopods are presented in Additional file 5: Figure S4. The most frequent amino acids in the PCGs of *T. chinensis* are Ile (10.34%), Leu2 (9.69%), Phe (8.92%) and Ser2 (7.86%), and in *I. japonensis*: Leu2 (10.96%), Ile (10%), Phe (8.41%) and Met (7.97%). In *T. chinensis*, the most frequent codons were ATT (isoleucine, 7.89%), TTA (leucine, 7.86%) and TTT (phenylalanine, 7.15%), whereas the GCG codon for alanine only appeared once. For *I. japonensis*, the most frequent codons were TTA (leucine, 8.71%), ATT (isoleucine, 7.42%) and ATA (methionine, 6.71%), whereas the CTG codon for leucine and CGG for arginine were the least frequent codons, both appearing only twice. Relative synonymous codon usage values in the mt genomes of *T. chinensis* and *I. japonensis* reflected a significant bias towards A and T nucleotides (Additional file 5: Figure S4).

### Transfer RNA genes

We could only identify 21 tRNAs in the mt genome of *T. chinensis* (*trnS*1 appears to be missing), ranging in size from 52 bp (*trnC*) to 69 bp (*trnQ*). In the mt genome of *I. japonensis*, we also only identified 21 tRNA genes (*trnI* missing), ranging in size from 51 bp (*trnE*) to 66 bp (*trnS*1). Concatenated lengths of all tRNA genes in the two mt genomes were 1292 and 1251 bp, with A+T contents of 76.4% and 75.4%, respectively. Eleven tRNAs in *T. chinensis* and 13 in *I. japonensis* are encoded on the H-strand, while the remaining tRNAs are encoded on the L-strand.

### Ribosomal RNA genes

**Fig. 2:**
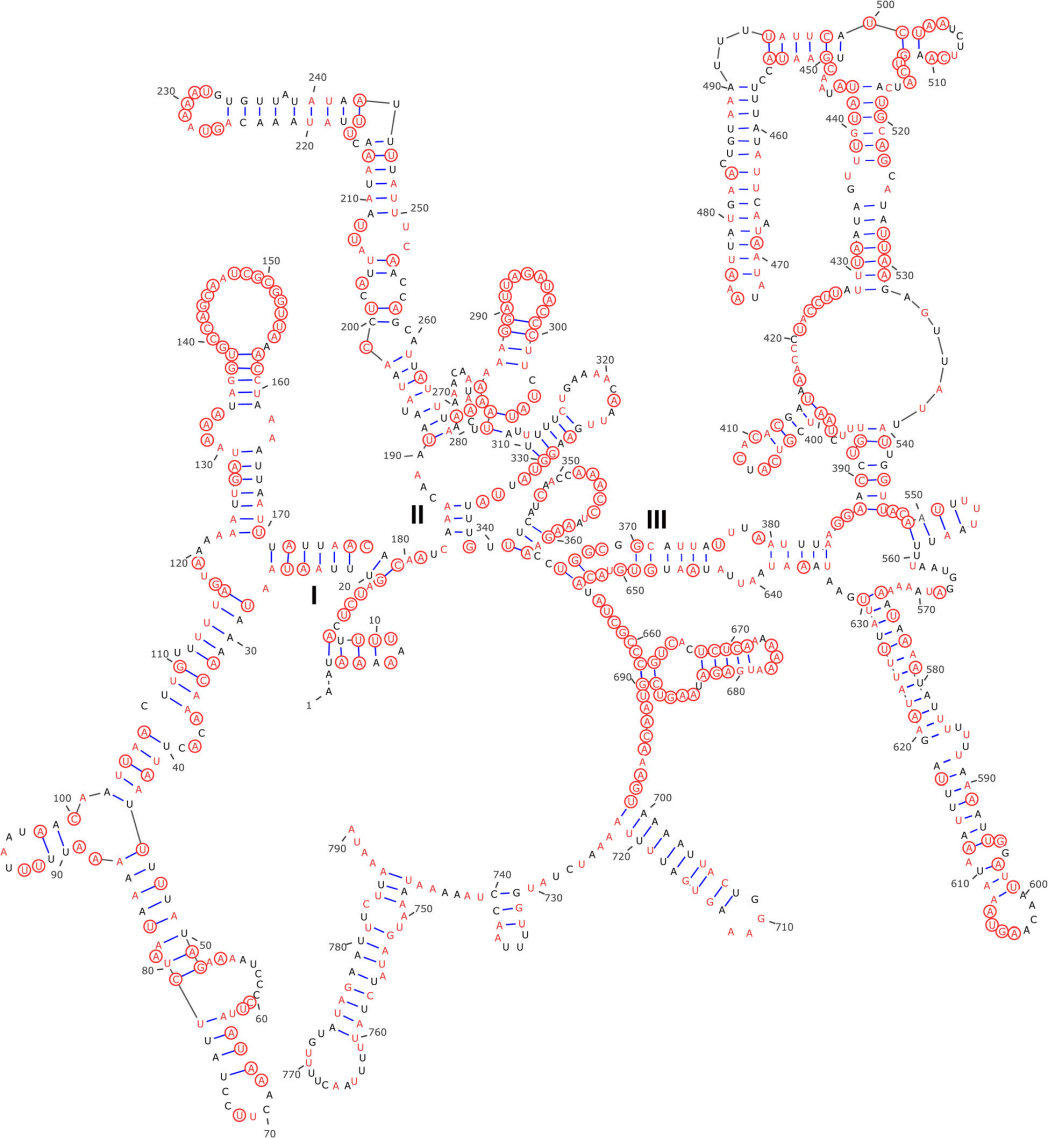
Predicted secondary structure of the *Tachaea chinensis* mitochondrial *rrnS* gene. Positions conserved between *T. chinensis* and *Ichthyoxenos japonensis* are colored in red. Positions conserved among the three species of the Cymothooidea, *T. chinensis*, *I. japonensis* and *Eurydice pulchra*, are circled in red. Different domains are labelled with Roman numerals

**Fig. 3:**
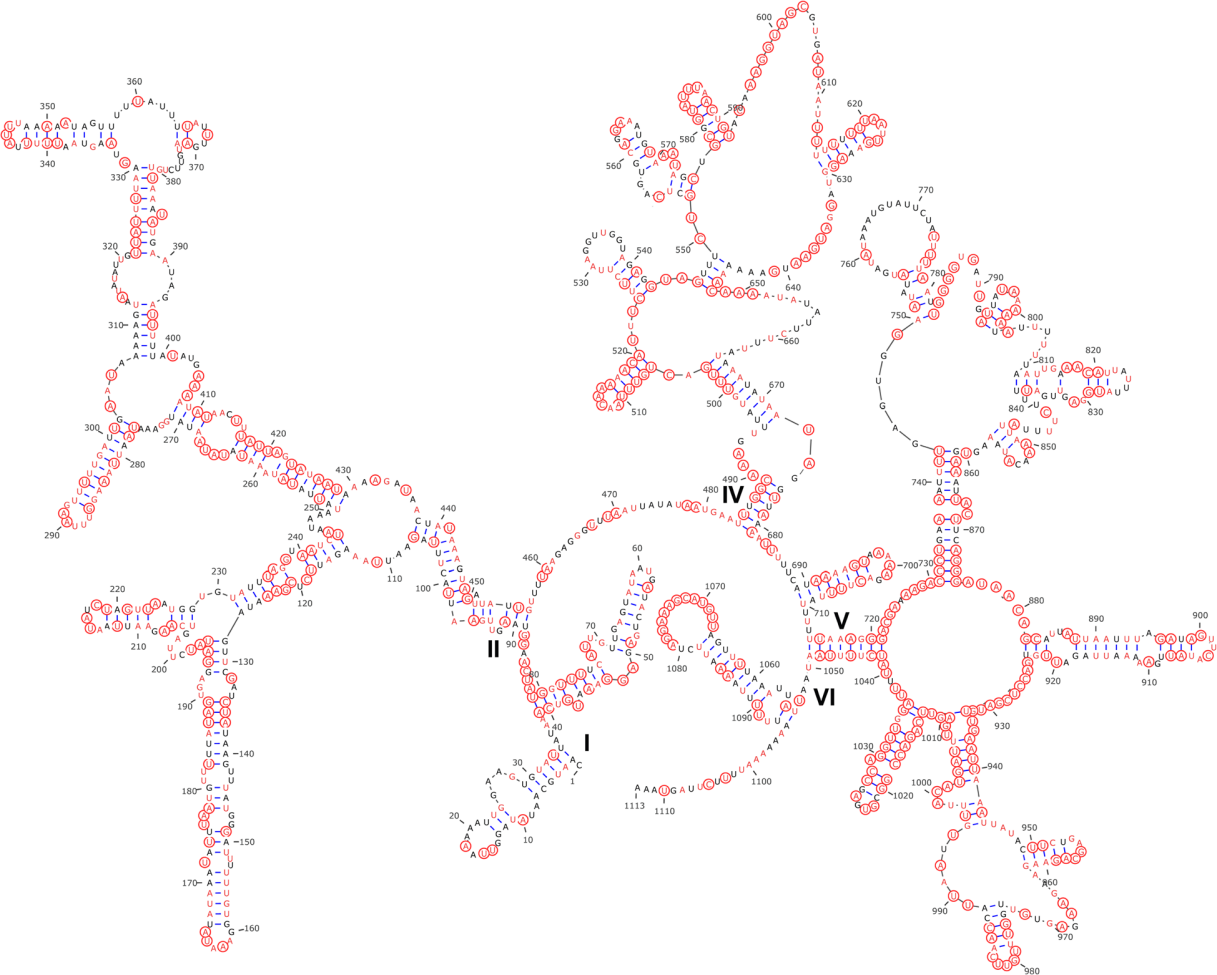
Predicted secondary structure of the *Tachaea chinensis* mitochondrial *rrnL* gene. Positions conserved between *T. chinensis* and *Ichthyoxenos japonensis* are colored in red. Positions conserved among the three species of the Cymothooidea, *T. chinensis*, *I. japonensis* and *Eurydice pulchra*, are circled in red. Different domains are labelled with Roman numerals

The lengths of rRNA genes are relatively similar in *T. chinensis* and *I. japonensis*: the large ribosomal subunit (*rrnL*) is 1113 and 1076 bp, respectively, whereas the small ribosomal subunit (*rrnS*) is 790 bp and 800 bp, respectively. Among the published isopod mt genes, the *rrnL* sequences ranged from 600 bp (*Armadillidium vulgare*) to 1367 bp (*L. quadripunctata*), while the *rrnS* sequences ranged from 687 bp (*A. vulgare*) to 850 bp (*L. oceanica*). The *rrnL* gene of *T. chinensis* and *I. japonensis* is found between the *trnS*2 and *trnQ* genes. The *rrnS* gene, however, is found in different locations: between *trnN* and *trnI* in *T. chinensis*, but between *trnN* and the control region in *I. japonensis*. This might be merely an annotation artefact caused by our failure to identify the *trnI* gene in the latter species. Predicted secondary structures of *rrnS* and *rrnL* genes of *T. chinensis* (used as a representative for Isopoda) are shown in Figs. 2 and 3, respectively. The structures of the two rRNA genes in *T. chinensis* generally resemble orthologs in other crustaceans. Secondary structures of *rrnS* and *rrnL* genes contain three and five domains, respectively. The domain III of *rrnL* is absent (this gene normally possesses six domains), which was also reported in other arthropod *rrnL* genes [71, 72]. Multiple alignment of of *rrnS* sequences of three species of the Cymothooidea (*T. chinensis*, *I. japonensis* and *E. pulchra*) comprised 857 positions, 341 of which were conserved (39.8%), with domain III being the most conserved region (42.2%).

Multiple alignment of the *rrnL* gene of the three species of the Cymothooidea comprised 1189 positions, 501 of which were conserved (42.1%). Domains I, II and VI were highly variable, with only 25.8–39.3% conserved positions, while domains IV and V were more conserved, with 49.4 and 52.4% conserved positions (Fig. 3). We also found that rRNA genes were more conserved between *T. chinensis* and *I. japonensis* (*rrnS*, 61.4%; *rrnL*, 66.8%) than between *T. chinensis* and *E. pulchra* (42.4 and 49.2%, respectively).

### Non-coding regions

**Fig. 4:**
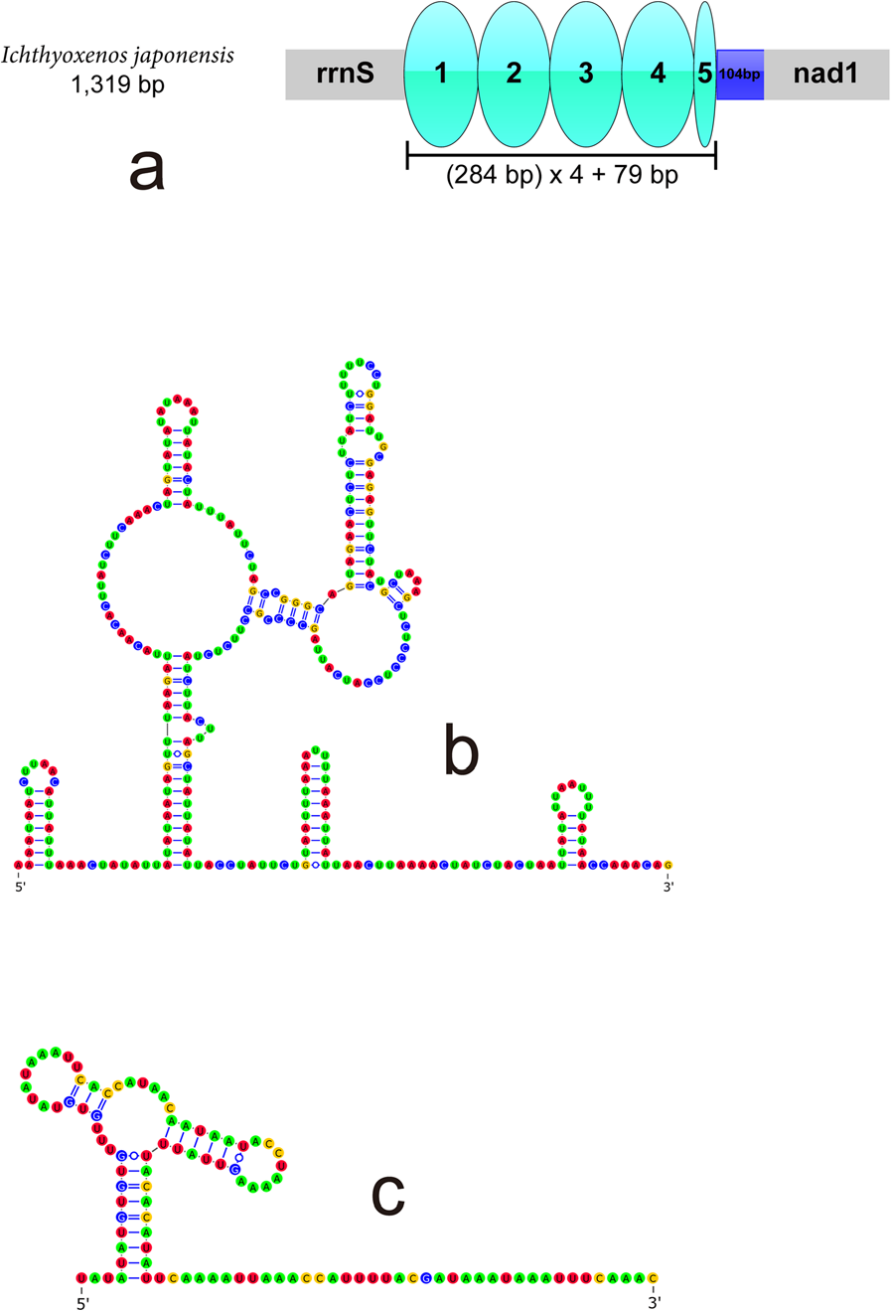
Organization and secondary structure of the major non-coding region of *Ichthyoxenos japonensis*. **a** Organization of the major non-coding region of *I. japonensis*. Turquoise ovals indicate tandem repeats in the repetitive region, and the blue box indicates the non-repetitive region. **b** Secondary structure of the consensus repeat unit (repeat 1) of the repetitive region. **c** Secondary structure of the non-repetitive region

We identified eleven short intergenic regions (1–60 bp) interspersed within the mt genome of *T. chinensis*, adding up to a total of 178 bp (Table 2). The control region (CR) is 322 bp in size and located between *trnI* and *trnE*. In addition, we could not identify any repetitive sequences in the CR of *T. chinensis*. In the mt genome of *I. japonensis*, we identified nine short intergenic regions (1–56 bp; Table 1), adding up to 97 bp. The CR (1319 bp) was identified between *rrnS* and *nad*1. Within the CR there is a highly repetitive region (HRR): a 284 bp sequence tandemly repeated four times, with a partial fifth repeat (Fig. 4). Repeat units 2 and 4 are identical in nucleotide composition (both 284 bp). In comparison to these two repeat units, units 1 and 3 differ in one nucleotide. Repeat unit 5 shared the same SNP as unit 1, but it was considerably truncated (79 bp), with approximately 205 bp missing at the 3′-end. Stem-loop secondary structure of the consensus repeat unit is presented in Fig. 4. The adjacent downstream sequence (not containing repeats) can also be folded into two stem-loop structures (Fig. 4).

### Gene translocations

**Fig. 5:**
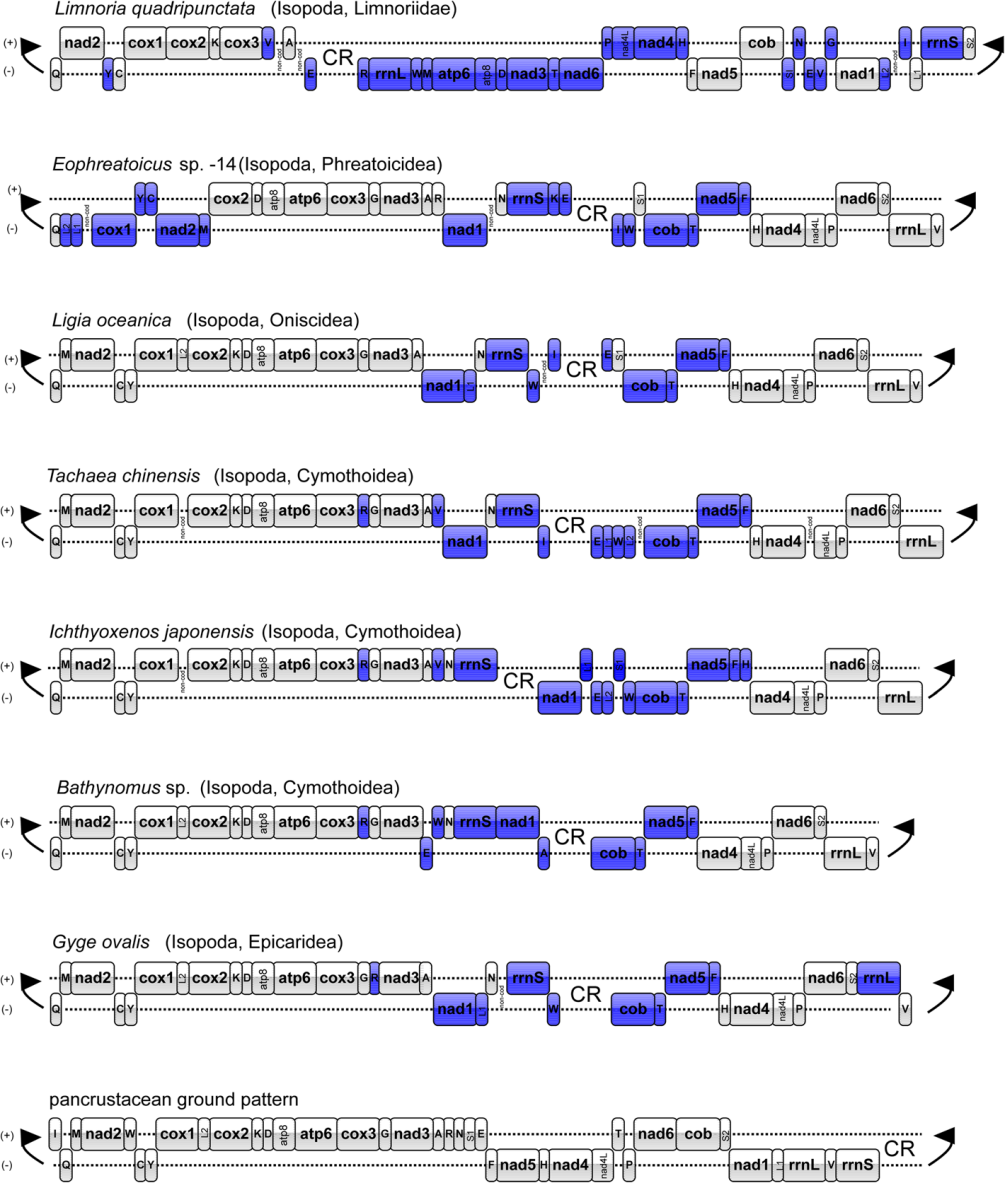
Comparison of mitochondrial gene arrangements of six complete isopod mt genomes and the putative pancrustacean ground pattern. Colored genes mark translocated genes in comparison to the ground pattern

Both newly-sequenced species (*T. chinensis* and *I. japonensis*) possess unique gene order (compared to other isopods) (Additional file 6: Figure S5), exhibiting rearrangements (relative to the pancrustacean ground pattern) in *rrnS*, *nad*1, *cob*, *nad*5 and nine tRNA genes (Fig. 5). Among the latter, *trnR*, *trnV*, *trnE*, *trnL*1, *trnW*, *trnL*2, *trnT* and *trnF* were rearranged in both species, whereas *trnI* rearrangement was unique to *T. chinensis*, and *trnS*1 to *I. japonensis*. Gene order was conserved between these two species except for the location of *nad*1 and five tRNAs: *trnL*1, *trnS*1 (not found in *T. chinensis*), *trnI* (not found in *I. japonensis*), *trnW* and *trnH*. *nad*1 was located between *trnV* and *trnN* in *T. chinensis* (and a few other isopods), but in *I. japonensis* it was found between the CR and *trnL*1.

### Phylogenetic analyses

**Fig. 6:**
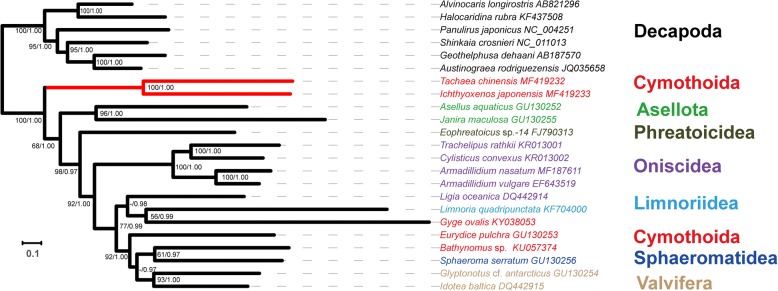
Phylogenetic tree for isopod mt genomes and six outgroup species inferred using BI and ML analyses based on concatenated nucleotide sequences of 13 protein-coding genes. Numbers next to nodes indicate bootstrap percentages of the RAxML analysis (left) and Bayesian posterior probabilities (right), where “-” indicates that bootstrap value was below 50%. Species names are colored by suborder (shown to the right). The scale-bar indicates evolutionary distance (substitutions per site)

Both phylogenetic trees (BI and ML; based on nucleotide sequences of 13 PCGs) had identical topologies (Fig. 6). *Tachaea chinensis* and *I. japonensis* formed a sister-clade to all other isopods included in the analysis, with a maximum nodal support (BP = 100, BPP = 1). The other parasitic species of the Cymothoida, *G. ovalis*, formed a clade with the single species of the Limnoriidea, *L. quadripunctata*, and then clustered together with *L. oceanica* a species of the Oniscidea. The two free-living species of the Cymothoida were found in the derived portion of the phylogram; *Bathynomus* sp. formed a sister clade with the single species of the Sphaeromatidea (*S. serratum*), and then grouped with a monophyletic Valvifera (*G.* cf. *antarcticus* and *I. baltica*). *Eurydice pulchra* formed a sister-clade with this entire clade (*Bathynomus* sp. + Sphaeromatidea + Valvifera). Apart from the Cymothoida and Oniscidea, the remaining major isopod suborders were monophyletic.

## Discussion

### Mt genome organization and base composition

Both mt genome sequences studied here had high A+T content (*T. chinensis*: 72.7%; *I. japonensis*: 72.8%). This is higher than in the remaining five complete isopod mt genomes: 58.7% in *Bathynomus* sp.; 59.6% in *G. ovalis*; 60.9% in *L. oceanica*; 66.3% in *L. quadripunctata*; and 69.6% in *Eophreatoicus* sp. (Additional file 7: Table S2). The A+T content varied profoundly between RNAs (77.6% in rRNAs, 76.4% in tRNAs in *T. chinensis*; 75.5% in rRNAs, 75.4% in tRNAs in *I. japonensis*) and PCGs (71.2% in *T. chinensis* and 71.7% in *I. japonensis*). This trend is shared by the remaining sequenced isopod mt genomes.

### Protein-coding genes and codon usage

Similar frequencies of the most frequent amino acids in *T. chinensis* and *I. japonensis* were also observed in other isopod species. For example, Leu2 is the most frequent amino acid in the PCGs of the other eight isopods, followed by Phe in four species. However, in *Eurydice pulchra* and *Bathynomus* sp., the other two sequenced cymothooidean free-living isopods, the most frequent amino acids are notably different from the two studied sequences: Gly, Val, Leu1, and Leu2. In *G. ovalis*, the other sequenced cymothooidean parasitic isopod, the most frequent amino acids are Leu1, Val, Gly and Phe. In addition, Cys is the least used codon in all published isopods, with the utilization rate less than 1% in 14 of 17 species. Almost all of the most frequently used codons ended with A/T, which is reflected in the high A+T bias in isopod mt genomes.

### Transfer RNA genes

**Fig. 7:**
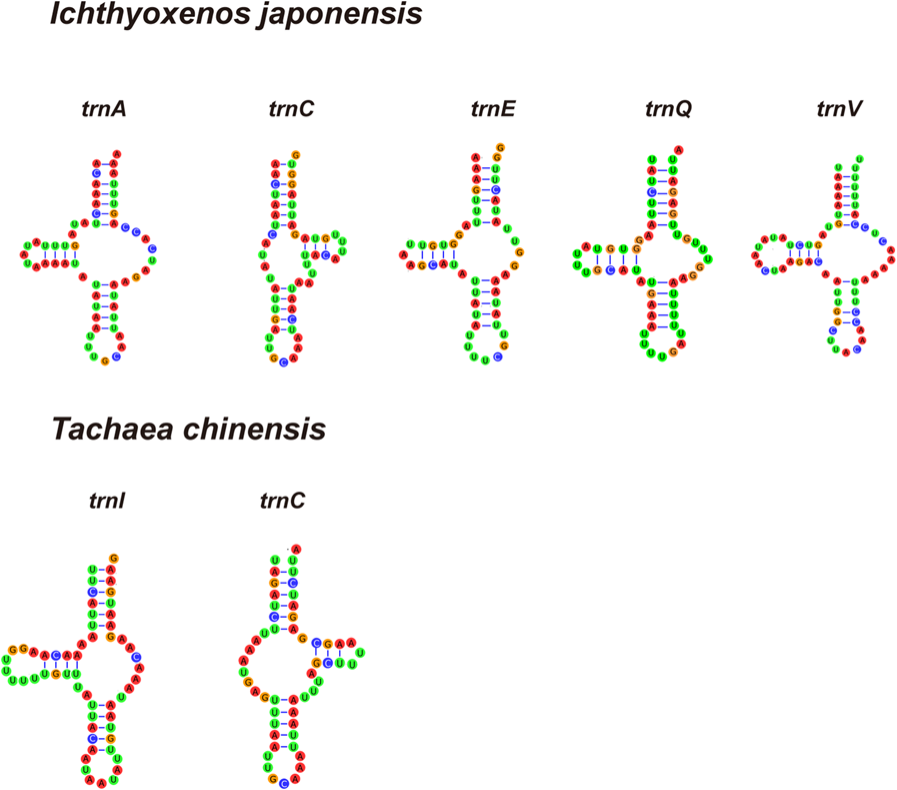
tRNAs of *Tachaea chinensis* and *Ichthyoxenos japonensis* exhibiting a non-standard secondary structure

Most identified tRNAs could be folded into the standard cloverleaf structure, with the exception of five tRNAs that lacked the T*ψ*C arm (*trnA*, *trnE*, *trnQ* and *trnV* in *I. japonensis*, and *trnI* in *T. chinensis*) and *trnC*, which lacked the DHU arm in both species (Fig. 7). All tRNAs had the standard anti-codons for Arthropoda, except *trnI* in *T. chinensis*, which used the AAT anticodon.

### Ribosomal RNA genes

Comparative analyses of the secondary structure of *rrnS* among three species of the Cymothooidea (*T. chinensis*, *I. japonensis* and *E. pulchra*) showed uneven distribution of conserved nucleotides, with domain III being the most conserved region. Multiple alignment of the *rrnL* gene of the three species showed that domains IV and V were more conserved, which is similar to the conservation pattern reported in amphipod *rrnL* genes [72]. We also found that rRNA genes were more conserved between *T. chinensis* and *I. japonensis* than between *T. chinensis* and *E. pulchra*, which indicates a closer relationship between *T. chinensis* and *I. japonensis*.

### Non-coding regions

The location of the control region (CR) in *T. chinensis* is similar to that of the CR of *L. oceanica* and *Eophreatoicus* sp. Compared to the CRs of other published isopod species, which range from 410 bp (*Eophreatoicus* sp.) [36] to 1525 bp (*L. quadripunctata*) [39], it is relatively short. We did not identify any repetitive sequences in the CR of *T. chinensis*, as opposed to the CR of another isopod, *L. oceanica*, which contains repetitive sequences with high variability in length and number of repetitions [38]. Stem-loop secondary structure of the consensus repeat unit and the adjacent downstream sequence were found within the CR of the mt genome of *I. japonensis*. Similar stem-loop structures were found in other crustacean species, such as the mantis shrimp *Squilla mantis*, the spiny lobster *Panulirus japonicus*, the common sea slater, *L. oceanica* [38], and the holoparasitic isopod *G. ovalis* [37], so we suspect that this structure may be associated with the origin of replication [73].

### Gene translocations

Among the 15 studied isopod mt genomes, each possessed a unique arrangement, but differences were mostly limited to the position of tRNA genes or a few PCGs (Additional file 6: Figure S5). The gene order of isopod mt genomes generally differs from the pancrustacean ground pattern [36, 38, 39, 74], and the two studied mt genomes are no exception in this aspect. When compared with the pancrustacean ground pattern, major translocations in isopods can mostly be localized to a highly variable “rearrangement hotspot” region around the CR [30], between *trnV* and *nad*4 (Fig. 5). Except for this region, gene orders of *I. japonensis* and *T. chinensis* are identical, and highly conserved in relation to the majority of published isopod mt genomes.

### Phylogenetic analyses

Under the currently prevalent classification of isopods, the Phreatoicidea is considered to be the basal group, whereas the Cymothoida is the most derived [11, 15, 75]. However, our results indicate that two parasitic species of the Cymothoida, *T. chinensis* and *I. japonensis*, form the basal isopod group. This topology is inconsistent with phylogenetic relationships inferred from morphological data [10, 15, 16] but is supported by some other molecular phylogenetic analyses relying on *18S* and combined nuclear and mitochondrial sequences [15, 17, 18]. Brusca & Wilson [10] suggested that the Phreatoicidea is the basal group, and the Cirolanidae, Corallanidae and Cymothoidae are highly derived within the isopods. Wilson [15] evaluated the relationships of isopods, relying on combined *18S* and morphological datasets; *18S* data suggested the basal position for the Asellota, whereas the two included species of the Phreatoicidea were among the derived isopod species. However, the combined analysis resolved the parasitic isopod groups Bopyroidea, Gnathiidae and Cymothoidae as basal isopod groups. Finally, a very recent phylogenetic analysis based on nuclear (*18S* and *28S*) and mitochondrial (*cox*1) genes also found that three parasitic species of the Cymothoida and a single species of the Sphaeromatidea formed the basal clade of isopods [18].

Phylogenetic analyses of nine and twelve nearly complete isopod mt genomes indicated that Phreatoicidea (and Asellota) clades form a sister clade to all other isopods [30, 35]. However, a recent analysis of a mt genome dataset included sequences from 17 isopod species and found that the Limnoriidae is the basal clade, followed by Asellota, while Phreatoicidea was placed centrally in the isopods [18]. The most recent analysis of mt genome sequences of 12 isopod species [37] resolved Asellota as the basal clade, followed by the Phreatoicidea, with the holoparasitic *G. ovalis* placed centrally in the cladogram and not clustering with other cymothoid taxa.

Brandt & Poore [5] conducted a cladistic analysis with morphological characters to explore relationships within the Flabellifera (now accepted as Cymothoida and Sphaeromatidea), and suggested that the Cymothoida comprised two monophyletic clades. They also suggested that the Cirolanidae should be elevated to a superfamily rank, the Anthuridea be treated as a superfamily, and the Epicaridea as two superfamilies.

Our results question the monophyly of the Cymothoida, with a deep evolutionary split between the two free-living species (*E. pulchra* and *Bathynomus* sp.) + one parasitic species (*G. ovalis*) and the two newly-sequenced parasitic Cymothoida species (disregarding the *L. quadripunctata* issue discussed above). Wilson [15] suggested that monophyly of the Cymothoida is supported by morphological data (202 characters, 52 isopods and 23 other malacostracans), but rejected by molecular data (*18S*); indeed, monophyly of the Cymothoida has been rejected by several phylogenetic analyses based on molecular data [15, 18, 35, 37]. For example, *18S* data produced a topology where several parasitic cymothoids formed a clade external to the main isopod clade [15]; in a combined nuclear (*18S* and *28S*) and mitochondrial (*cox*1) dataset, three parasitic cymothoids and a single species of the Sphaeromatidea formed the basal isopod clade, followed by a clade including 11 species of the Cymothoida, whereas free-living species of the Cymothoida clustered with five species of the Oniscidea [18]. Mitochondrial phylogenomic analysis of 12 isopod species [37] revealed that the holoparasitic *G. ovalis* did not cluster with the other two free-living cymothoid species. Furthermore, even a combined morpho-molecular dataset produced a deep split between the free-living and parasitic cymothoidans [15].

In addition, although both *E. pulchra* and *Bathynomus* sp. belong to the Cirolanidae, they did not form a monophyletic clade. A paraphyletic Cirolanidae was also produced by two other studies based on mitogenomic data [18, 35] and a study based on combined mito-nuclear molecular markers [18].

Except for the separation of parasitic and free-living groups in the Cymothoida, another noteworthy finding is the phylogenetic position of the holoparasitic epicaridean *G. ovalis*. Both nuclear (*18S*) [75] and morphological [5] data usually produce closely related positions of the Epicaridea and Cymothoidae. However, different phylogenetic position of the Epicaridea within Isopoda were proposed. Dreyer & Wagele [11] suggested that epicarideans evolved from species that parasitized fishes (Cymothoidae) and should be considered a family-level taxon within the superfamily Cymothooidea. Brandt & Poore [5] proposed that Epicaridea should be recognized as two superfamilies, Bopyroidea and Cryptoniscoidea, within the suborder Cymothoida. Boyko et al. [21] found that *18S* data supported retaining Epicaridea as a taxon within the Cymothoida, but distinct from the Cymothooidea. A dataset comprised of mt genome sequences of 12 isopod species, supported the Epicaridea (represented by *G. ovalis*) as a suborder [37]. However, our study suggests that *G. ovalis* is placed centrally in the cladogram, where it clusters with the Limnoriidea, and not with either the basal parasitic clade (families Cymothoidae and Corallanidae) or the other free-living cymothoid taxa (family Cirolanidae). However, as both species (*G. ovalis* and *L. quadripuncata*) exhibit rather long branches, a possibility of a long-branch attraction artifact should not be excluded here.

Therefore, this study indicates that the evolution and monophyly of the Cymothoida are not resolved. The relationships of parasitic and free-living groups require deeper attention, with more data and further analyses on a broader scale. Availability of a larger number of molecular data (mt genomes and nuclear genes) of parasitic and free-living isopods might confer a sufficiently high phylogenetic resolution to enable us to resolve their relationships, and ultimately allow us to better understand the evolutionary history of the entire Isopoda.

In addition, it should be noted that we did not recover the Oniscidea as monophyletic. Several phylogenetic analyses relying on whole mt genome data also found that *L. oceanica* is more closely related to non-oniscid taxa [18, 30, 35, 37]. Moreover, recent nuclear *18S* [46] and mito-nuclear analyses [18] also presented evidence that the Oniscidea is not a monophyletic group.

Another aspect of isopod evolution that scientists are paying attention to is the expansion of the Cymothoidae into freshwater habitats. When discussing the habitat expansion, the acquisition and shifts of attachment modes must be considered as well, because it is considered that parasitic isopods always expand into new habitats by changing their attachment modes and hosts [25]. Smit et al. [4] suggested that Cymothoidae may have originated in the Jurassic era, on the basis of the existence of fossils of bopyrid isopods dated to that period, as this family (Bopyridae) is closely related to the Cymothoidae [21]. Recently, two fossil records of early cymothoid isopods dated to the Jurassic also supported this viewpoint [23, 47]. Hata et al. [25] suggested that cymothoids may have originated in deep seas, subsequently expanded to shallow seas, and then to brackish and/or freshwater, by shifting host species. Invasion of freshwater habitats may have occurred independently several times [22, 25]. The freshwater habitat and the burrowing habit may be correlated within the Cymothoidae [22]. However, the availability of molecular data for freshwater and burrowing cymothoid species is limited to two freshwater species, a burrowing parasite (*Artystone* sp.) and a buccal parasite (*Ichthyoxenos tanganyikae*). Therefore, more molecular data are needed to define the timing and frequency of evolution of freshwater and abdominal cavity-burrowing parasites [25].

## Conclusions

In this study we sequenced the mt genomes of two parasitic cymothoid isopods, *T. chinensis* and *I. japonensis*, and conducted a comparative analysis using the available isopod mt genomes. Among the 17 compared isopod mt genomes (including the two studied species), each has a unique gene arrangement, which signifies high variability of gene order in the Isopoda. The two parasitic cymothoids examined here clustered together at the base of the isopod phylogram, forming a sister group to all remaining (available) isopods, which challenges the traditional phylogeny of this order. Moreover, parasitic and free-living Cymothoida formed evolutionary distant clades, and *G. ovalis* did not cluster with the two parasitic species of the Cymothoidae. The mt genome sequences of *T. chinensis* and *I. japonensis*, as representatives of micropredators and burrowing parasites in freshwater habitats, present a useful resource for further evolutionary and taxonomic studies of the Cymothoida, especially the phylogenetic position of epicarideans, the evolution of different life-styles, the evolution of parasitic strategies, invasion of freshwater habitats and the classification and identification of the genus *Ichthyoxenos*. Due to the current shortage of molecular data carrying a sufficient amount of phylogenetic information, the relationships of the Epicaridea, Cymothooidea, Limnoriidea, Valvifera and Spharomatidea, as well as the monophyly of the Cymothoida and Oniscidea, remain unresolved. In order to resolve these phylogenetic questions, future studies should aim to sequence a much larger number of isopod mt genomes and nuclear genes across a broad range of isopod taxa.

## Additional files

Additional file 1: **Figure S1.** Maximum likelihood tree for the family Cymothoidae based on mitochondrial *16S* rRNA sequence data conducted in MEGA7. (TIF 5180 kb)

Additional file 2: **Figure S2.** An image of *Tachaea chinensis*. (TIF 6612 kb)

Additional file 3: **Figure S3.** An image of *Ichthyoxenos japonensis*. (TIF 5659 kb)

Additional file 4: **Table S1.** Primers used to amplify and sequence the mitochondrial genomes of the two parasitic cymothoid isopods *Ichthyoxenos japonensis* and *Tachaea chinensis. (XLSX 13 kb)*

Additional file 5: **Figure S4.** Relative synonymous codon usage of mitochondrial genomes of 17 isopods. Codon families are labelled on the x-axis. Values on the top of the bars refer to amino acid usage. (TIF 570 kb)

Additional file 6: **Figure S5.** Comparison of mitochondrial gene arrangements of 17 isopod mt genomes. (TIF 1590 kb)

Additional file 7: **Table S2.** Comparison of 17 isopod mitochondrial genomes. (XLSX 13 kb)

## Abbreviations

BI: Bayesian inference
CR: control region
ML: maximum likelihood
mt: mitochondrial
PCGs: protein-coding genes
rRNAs: ribosomal RNA genes
*rrnL*: large ribosomal subunit
*rrnS*: small ribosomal subunit
RSCU: relative synonymous codon usage
tRNAs: transfer RNA genes
