# Supplementary figures and images for: Basal position of two new complete mitochondrial genomes of parasitic Cymothoida (Crustacea: Isopoda) challenges the monophyly of the suborder and phylogeny of the entire order
Source: Parasit Vectors. 2018 Dec 10;11:628. doi: 10.1186/s13071-018-3162-4 (PMC6287365; doi:10.1186/s13071-018-3162-4)

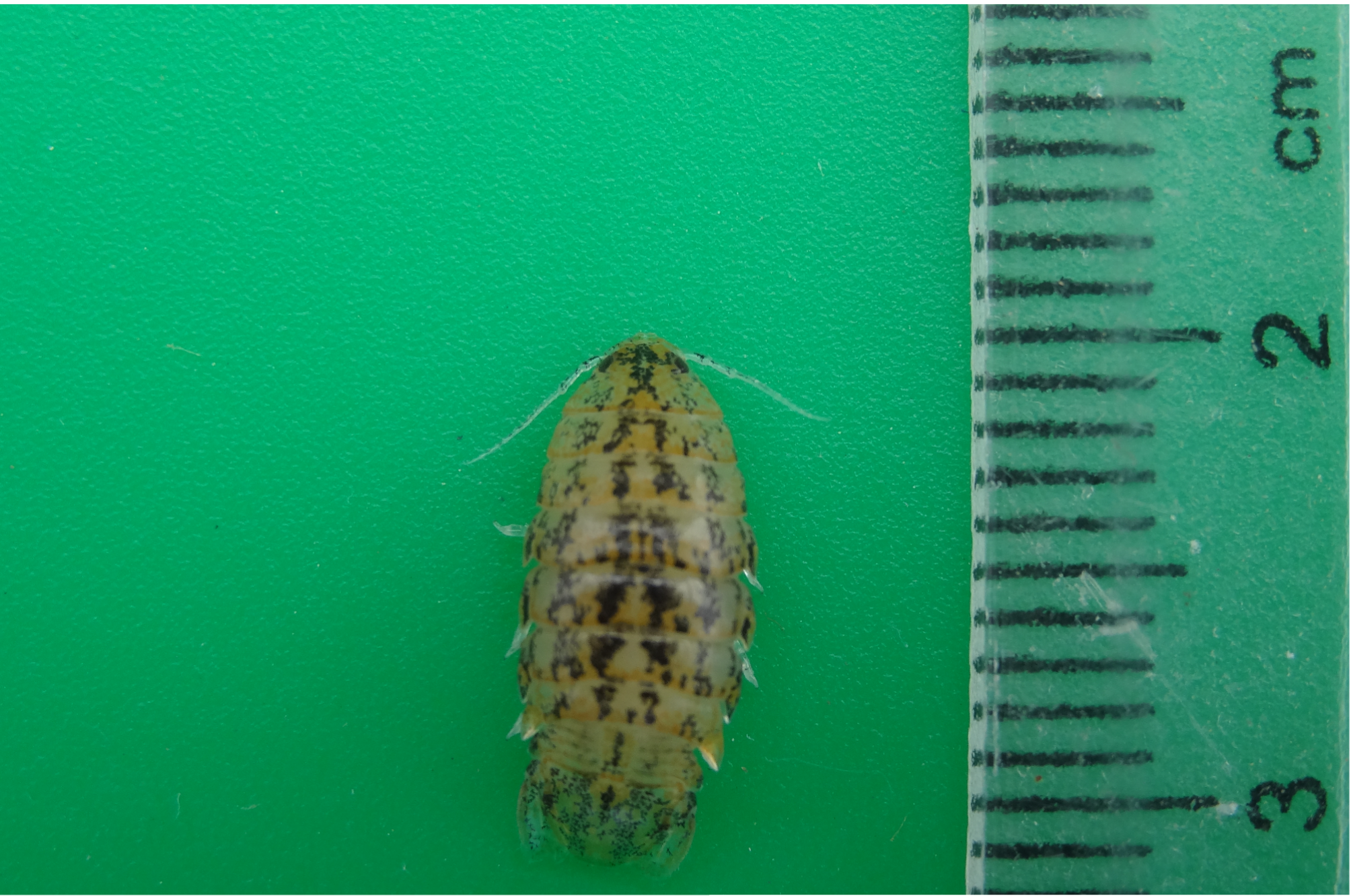

Supplement: Supplementary file 2 — Figure S2. An image of Tachaea chinensis. (TIF 6612 kb) [file 13071_2018_3162_MOESM2_ESM.tif]

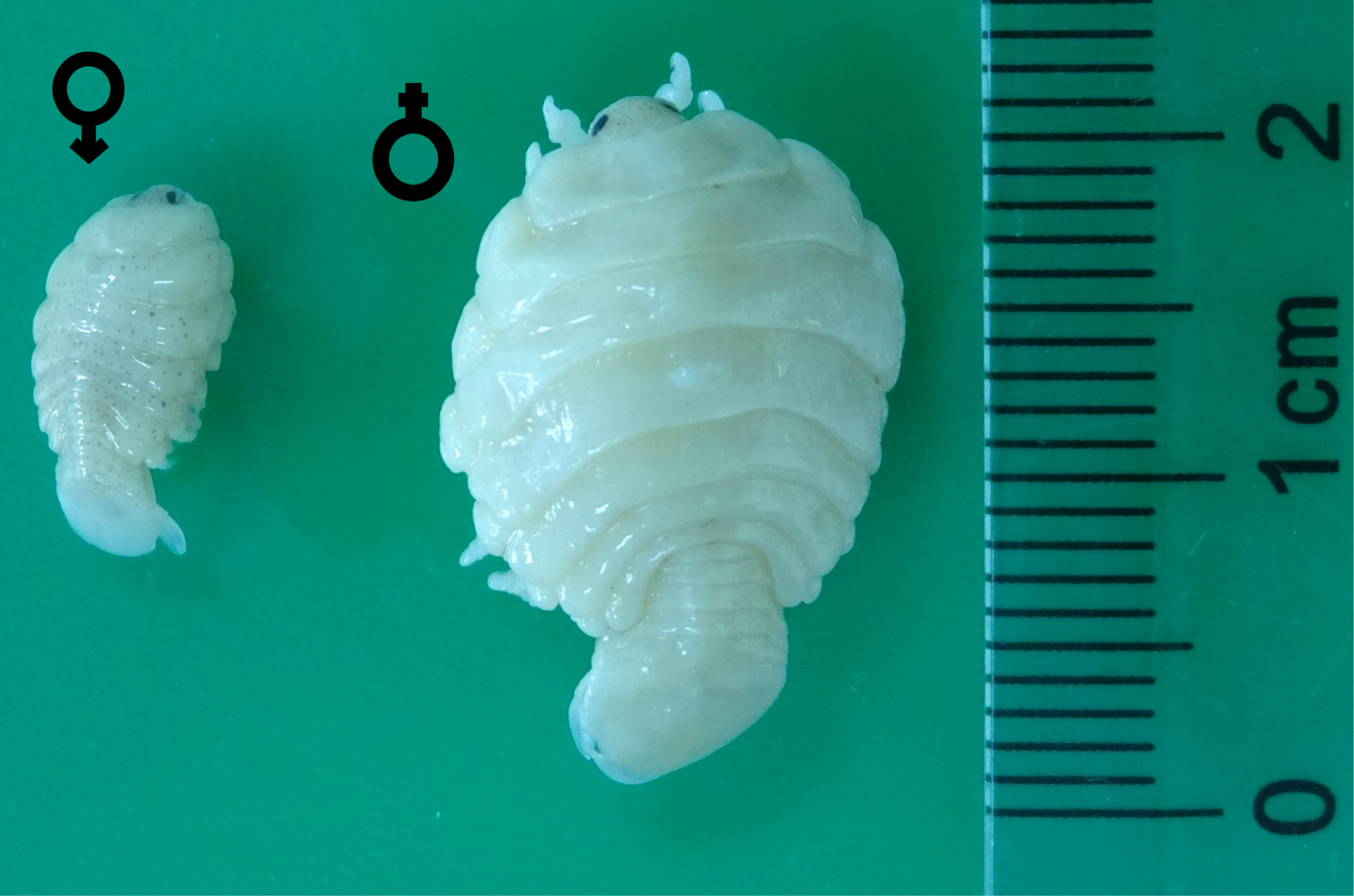

Supplement: Supplementary file 3 — Figure S3. An image of Ichthyoxenos japonensis. (TIF 5659 kb) [file 13071_2018_3162_MOESM3_ESM.tif]

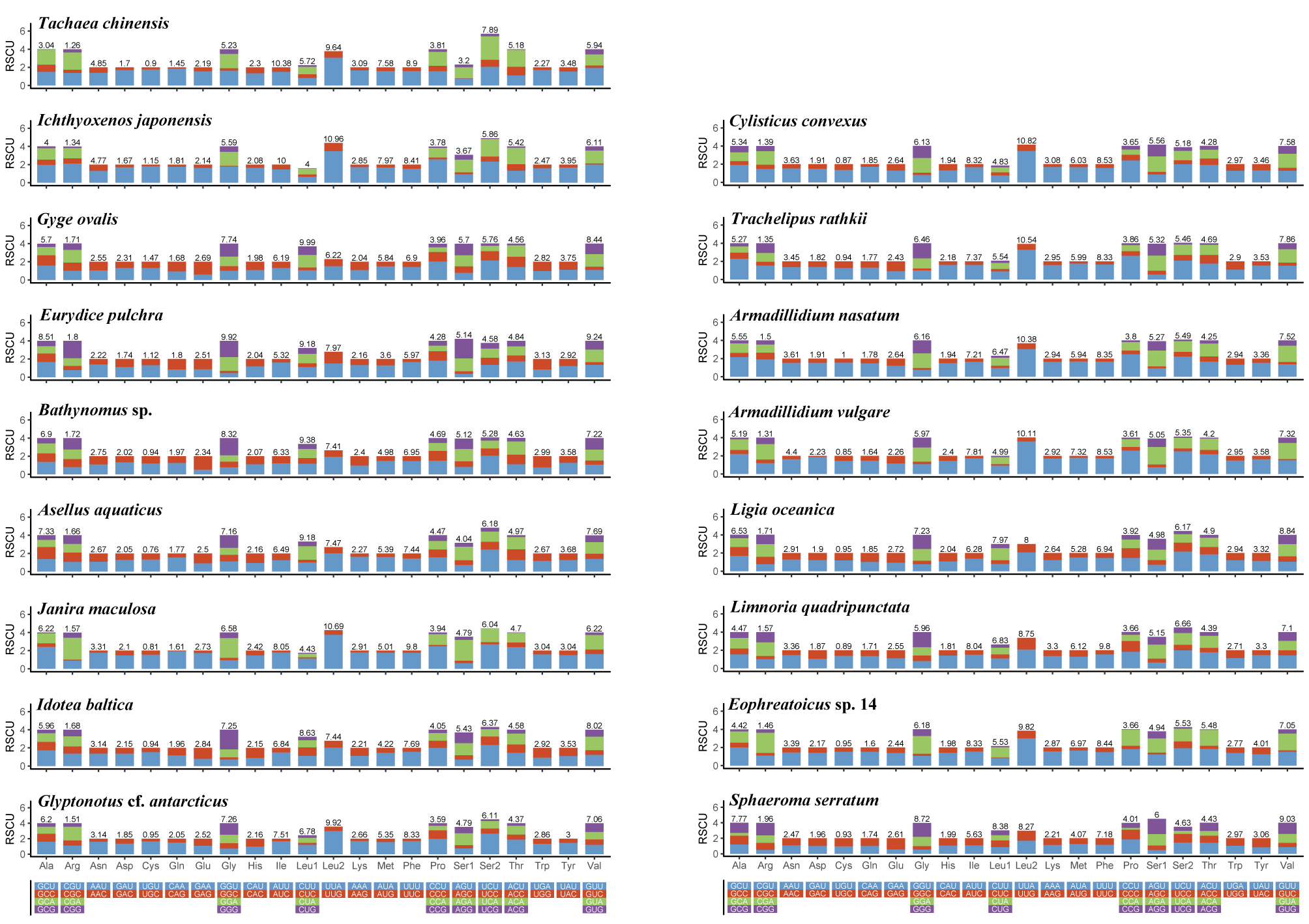

Supplement: Supplementary file 5 — Figure S4. Relative synonymous codon usage of mitochondrial genomes of 17 isopods. Codon families are labelled on the x-axis. Values on the top of the bars refer to amino acid usage. (TIF 570 kb) [file 13071_2018_3162_MOESM5_ESM.tif]

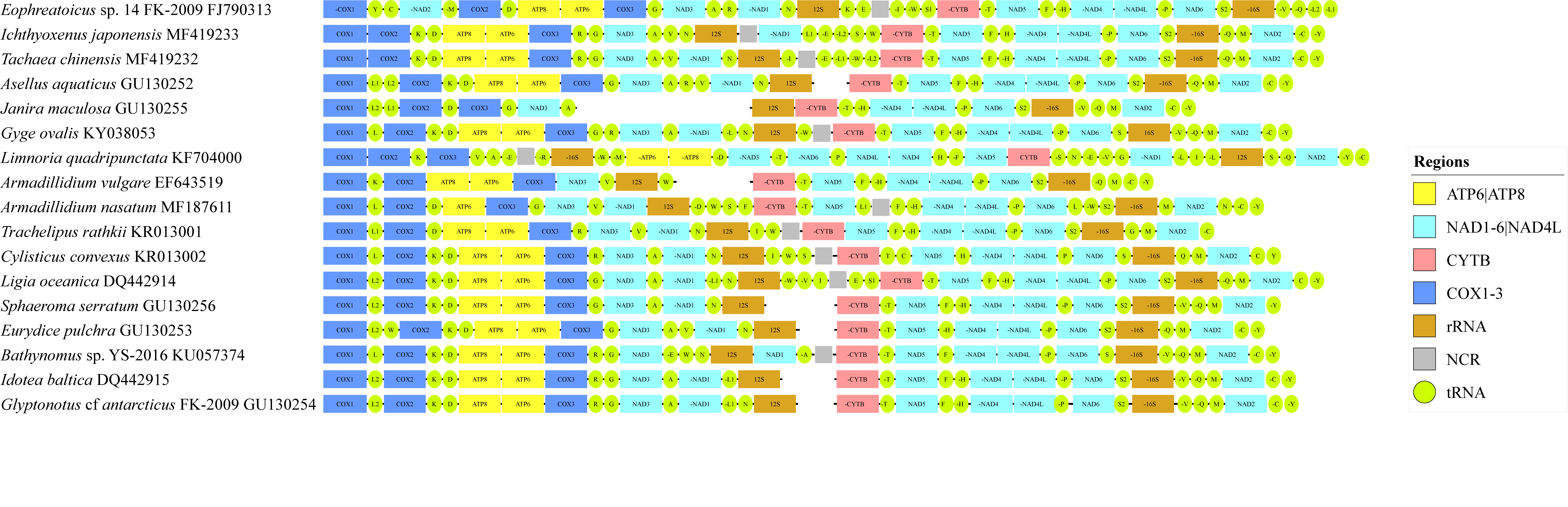

Supplement: Supplementary file 6 — Figure S5. Comparison of mitochondrial gene arrangements of 17 isopod mt genomes. (TIF 1590 kb) [file 13071_2018_3162_MOESM6_ESM.tif]
